# On the Effects of Blood as an Antidote for Arsenic

**Published:** 1845-08

**Authors:** 


*On the effects of Blood as an antidote to Arsenic.*—The desire of discovering in poisoning by arsenic, an antidote which could be obtained and employed under any circumstances, led the author to institute the following toxicological experiments: Arsenic having a great affinity to the constituents of blood, the author administered, at noon, to a well fed healthy dog, nine years of age, three grains of arsenious acid dissolved in diluted milk, after the animal had been eighteen hours without food; a quarter of an hour after, eighteen ounces of blood, taken from a calf just killed, were poured into its mouth. Considerable perspiration and trembling all over the body ensued, then thirst, dejection, and tendency to vomiting. At seven o'clock, P. M., the dog ate and drank freely, was lively, and apparently strong; neither vomiting, nor stool, nor urinary secretion ensued during the night. The following day the dog was left quiet. On the third day, six grains of arsenious acid were given in diluted broth, after fasting for twelve hours: within ten minutes twelve ounces of blood were poured in, the animal's struggles preventing a greater quantity from being administered. It drank a great deal of water. No other symptoms appeared than those perceived at the first experiment, viz: perspiration, exhaustion, and trembling. In the evening the dog was quite lively. A day's interval was allowed for his recovery, and on the fifth day he had nine grains of arsenious acid in diluted milk; after a few minutes, nine ounces of blood were poured into its mouth with great trouble. The symptoms were the same as on the former occasion, together with the singular fact, that a pterygium of the right eye, with which the eye was affected, contracted itself, and disappeared the next day; the right eye was then as clear as the left. On the seventh day the dog received twelve grains of arsenious acid given in broth, and no more than eight ounces of blood could be injected; the perspiration was so considerable, that the animal appeared as if it had been bathed; the thirst was great. The animal howled constantly, with a hoarse voice, and evacuated fæces and urine, which had not been the case in the former experiment. After the lapse of twenty-four hours, that is, on the ninth day, the dog had eighteen grains of arsenious acid, and about six ounces of blood. The dose was increased this time by six grains, because the author wished to close his experiments with the death of the dog. The effects were now very marked, great thirst, restlessness, convulsions, and complete prostration. At about eleven, P. M., the symptoms of poisoning had almost disappeared; after some days the dog recovered almost completely with the exception of hoarseness in barking. In order to ascertain the organic changes produced by the large quantities of arsenic which had been introdnced into the system, the dog was killed. Neither the pharynx nor fauces were inflamed nor spotted; the venous blood was gelatinous, the arterial coagulated and not quite red; the liver was very hard and fragile; the lungs were inflated, and covered with bluish spots in some places; they scarcely contained any blood; heart unaltered. The blood contained in the heart was gelatinous and black; this was particularly the case in the right venticle; stomach unaltered externally, thrown into deep folds, much inflamed internally; pylorus normal. Duodenum, ileum, and colon were also inflamed, and filled with digested food. In order to ascertain whether the arsenic had not entered the blood or the brain, the author collected the blood of the heart, evaporated it to dryness, powdered it, and mixed a part with an equal quantity of carbonate of potash, and half the quantity of coal powder, and, to his great surprise, he obtained two grains and a half of arsenic by sublimation. The other part of the dried blood was used for analysis by a liquid process, and arsenic was likewise obtained; the brain was also dried and powdered, and mixed with carbonate of potash, and again one grain and three quarters of arsenic were obtained; the liver, muscles, &c., would also have been analysed in a similar manner, if the body of the dog had not been taken away during the night, and thrown into the river without the author's knowledge. Though the experiments appear imperfect in consequence of the urine and perspiration not having been analysed, still the author suggests, in cases of poisoning by arsenic, to use the blood of a freshly killed animal as an antidote, if no medical treatment were readily attainable. Should the nauseous character of the remedy be a great objection, it would be greatly removed by the patient having his eyes bandaged while taking the blood.—*London Med. Times*, from *Franz. Apoiger in Buchner's Repertorium—Medical Examiner.*

